# Outcome and prognostic factors of *Pneumocystis jirovecii* pneumonia in immunocompromised adults: a prospective observational study

**DOI:** 10.1186/s13613-019-0604-x

**Published:** 2019-11-27

**Authors:** Benjamin Jean Gaborit, Benoit Tessoulin, Rose-Anne Lavergne, Florent Morio, Christine Sagan, Emmanuel Canet, Raphael Lecomte, Paul Leturnier, Colin Deschanvres, Lydie Khatchatourian, Nathalie Asseray, Charlotte Garret, Michael Vourch, Delphine Marest, François Raffi, David Boutoille, Jean Reignier

**Affiliations:** 1 Department of Infectious Diseases, Hôtel-Dieu University Hospital, University Hospital of Nantes and CIC 1413, INSERM, 1 Place Alexis-Ricordeau, 44000 Nantes, France; 2 Service d’Hématologie, University Hospital, INSERM, U1232, Université de Nantes, Nantes, France; 3 Laboratoire de Parasitologie-Mycologie, Institut de Biologie, University Hospital, Nantes, France; 4 INSERM, UMR1087, l’institut du thorax, Nantes, France; 5 Medical Intensive Care, University Hospital, Nantes, France; 6 EA 3826, Laboratory of Clinical and Experimental Therapeutics of Infections, IRS2-Nantes Biotech, Nantes, France

**Keywords:** *Pneumocystis jirovecii* pneumonia, Early prognostic score, High flow oxygen, Haematological malignancies, Alveolitis

## Abstract

**Background:**

*Pneumocystis jirovecii* pneumonia (PJP) remains a severe disease associated with high rates of invasive mechanical ventilation (MV) and mortality. The objectives of this study were to assess early risk factors for severe PJP and 90-day mortality, including the broncho-alveolar lavage fluid cytology profiles at diagnosis.

**Methods:**

We prospectively enrolled all patients meeting pre-defined diagnostic criteria for PJP admitted at Nantes university hospital, France, from January 2012 to January 2017. Diagnostic criteria for PJP were typical clinical features with microbiological confirmation of *P. jirovecii* cysts by direct examination or a positive specific quantitative real-time polymerase chain reaction (PCR) assay. Severe PJP was defined as hypoxemic acute respiratory failure requiring high-flow nasal oxygen with at least 50% FiO_2_, non-invasive ventilation, or MV.

**Results:**

Of 2446 respiratory samples investigated during the study period, 514 from 430 patients were positive for *P. jirovecii*. Of these 430 patients, 107 met criteria for PJP and were included in the study, 53 (49.5%) patients had severe PJP, including 30 who required MV. All patients were immunocompromised with haematological malignancy ranking first (*n* = 37, 35%), followed by solid organ transplantation (*n* = 27, 25%), HIV-infection (n = 21, 20%), systemic diseases (*n* = 13, 12%), solid tumors (*n* = 12, 11%) and primary immunodeficiency (*n* = 6, 8%). By multivariate analysis, factors independently associated with severity were older age (OR, 3.36; 95% CI 1.4–8.5; *p *< 0.05), a *P. jirovecii* microscopy-positive result from bronchoalveolar lavage (BAL) (OR, 1.3; 95% CI 1.54–9.3; *p *< 0.05); and absence of a BAL fluid alveolitis profile (OR, 3.2; 95% CI 1.27–8.8; *p *< 0.04). The 90-day mortality rate was 27%, increasing to 50% in the severe PJP group. Factors independently associated with 90-day mortality were worse SOFA score on day 1 (OR, 1.05; 95% CI 1.02–1.09; *p *< 0.001) whereas alveolitis at BAL was protective (OR, 0.79; 95% CI 0.65–0.96; *p *< 0.05). In the subgroup of HIV-negative patients, similar findings were obtained, then viral co-infection were independently associated with higher 90-day mortality (OR, 1.25; 95% CI 1.02–1.55; *p *< 0.05).

**Conclusions:**

Older age and *P. jirovecii* oocysts at microscopic examination of BAL were independently associated with severe PJP. Both initial PJP severity as evaluated by the SOFA score and viral co-infection predicted 90-day mortality. Alveolitis at BAL examination was associated with less severe PJP. The pathophysiological mechanism underlying this observation deserves further investigation.

## Background

Over the last 10 years, survival benefits provided by steady advances in antitumor chemotherapy and immunosuppressant regimens for patients with autoimmune diseases, haematological malignancies, and solid organ transplants have substantially increased the number of adults living with immunodeficiencies [1, 2]. Among opportunistic infections in immunocompromised adults, *Pneumocystis jirovecii* pneumonia (PJP) was associated with high rates of intubation and mortality [3]. Consequently, an early identification and optimal treatment of patients with PJP remains a key priority [4, 5].

Since the advent of antiretroviral therapy, the incidence and mortality rates of PJP among patients positive for the human immunodeficiency virus (HIV) have decreased steadily [6]. However, PJP is being increasingly diagnosed in HIV-negative patients, in whom it carries a poorer prognosis [7, 8]. A higher proportion of neutrophils in broncho-alveolar lavage (BAL) fluid during PJP was associated with higher risks of respiratory complications and mortality [9, 10]. In the same way, a low lymphocyte count in BAL fluid was a risk factor for the failure of trimethoprim/sulfamethoxazole (TMP/SMZ) therapy [11]. Early adjunctive steroid therapy for severe PJP dramatically decreased mortality rates in HIV-positive patients [12, 13] but had variable effects in their HIV-negative counterparts [14–16]. These findings suggest that the immunological status and underlying diagnosis may influence the pathophysiology of PJP and the risk of mortality [17, 18]. However, BAL fluid cytology profiles have not been adequately evaluated as potential prognostic factor and predictors of treatment responses.

The identification of early predictors of PJP outcomes, including BAL fluid findings, may help to determine which patients are most likely to benefit from intensive care and could justify adjunctive steroid therapy. The aim of this prospective study of patients with PJP was to identify early risk factors for severe PJP and 90-day mortality.

## Methods

### Study design and participants

This is a retrospective analysis of prospective cohort. From January 2012 to January 2017, all patients presenting an invasive fungal infection that has been diagnosed in our center have been included in a prospective registry (the French prospective surveillance programme, RESSIF network), thus PJP patients have been included in a sub-cohort. For each patient with a positive sample, the following clinical findings were prospectively investigated: dyspnea and/or cough in immunocompromised patients with interstitial syndrome by radiography or CT scan. Among all respiratory samples investigated for 6 years (*n* = 2446), all positive tests for Pneumocystis jirovecii samples (*n* = 430) were assessed by a biologist and a clinician to investigate criteria for PJP and include them in this prospective cohort. The following data were prospectively collected: age; sex; underlying disease; PJP prophylaxis; other medications including glucocorticoids taken during the past month; type of symptoms and symptom duration at PJP diagnosis and time from symptom onset to hospital admission. Secondary, the clinical data were collected for all patients from medical records: laboratory findings (white blood cell count; absolute neutrophil count; C-reactive protein [CRP] level; BAL fluid findings including the cell profile assessed by an independent cytologist on centrifuged BAL fluid samples prepared with the Wright-Giemsa and Perls stains to allow the determination of macrophage, lymphocyte, neutrophil, eosinophil, and basophil counts); presence of *P. jirovecii* and/or other fungi and/or bacteria and/or viruses (influenza viruses, respiratory syncytial virus, adenovirus, cytomegalovirus) were recorded at PJP diagnosis. The SOFA score on day 1 and the ratio of the arterial partial pressure of oxygen over the fraction of inspired oxygen (PaO_2_/FiO_2_) on day 1 [19] were recorded for each patient at PJP diagnosis. Anti-PJP medications, adjuvant glucocorticoid therapy, oxygen supplementation, and ventilatory support provided at admission were documented. Finally, patient outcomes including 90-day mortality were recorded. The collection of follow-up data ended in December 2017.

### Definitions

Severe PJP was defined as hypoxemic acute respiratory failure requiring high-flow nasal oxygen with at least 50% FiO_2_, non-invasive ventilation, or MV. Because not all patients were hospitalized in intensive care units, we chose this pragmatic and reproducible severity criterion. According to the Berlin definition [20], severe acute respiratory distress syndrome (ARDS) was defined as the presence of the following criteria within 3 days after ICU admission: new respiratory symptoms, bilateral opacities on chest radiographs or by CT, absence of suspected hydrostatic/cardiogenic pulmonary oedema, and PaO_2_/FiO_2_ ≤ 300. Lymphocytic alveolitis [21] was defined as a BAL fluid cell population containing more than 10% of lymphocytes and more than 5% of neutrophils combined with an activated macrophage phenotype, based on the diagnostic criteria for hypersensitivity pneumonitis, a condition characterised by alveolitis and migration to the alveoli of multiple cell types including activated T cells, monocytes, and natural killer cells [22]. Finally, patients with other pathogens associated with *P. jirovecii* in respiratory or blood samples were classified as having co-infection at ICU admission.

### Statistical analysis

Patient characteristics were described using mean ± SD for continuous variables (or 95% confidence interval [95% CI] when appropriate) and proportions for qualitative variables. Continuous variables were compared using the Wilcoxon rank-sum and Kruskal–Wallis tests and qualitative variables using Fisher’s exact test with computation of the odds ratios (ORs) and their 95% CIs. Overall survival was assessed using Kaplan–Meier curves and log-rank tests. Factors associated with severity were identified by logistic regression analysis. Factors associated with all-cause 90-day mortality were identified by logistic regression analysis of patients alive after day 90 versus patients who died before day 90. Only factors reaching statistical significance (*p* < 0.05), with no more than 10% missing data, were included in the multivariate model. When collinearity between co-variates was detected, only the variable with the highest OR was kept into the multivariate regression model. Results are reported as log-transformed coefficients with their 95% CIs. Missing data were not interpolated. Analyses were performed on the whole population, then excluding HIV+ patients.

## Results

### Patient characteristics

**Fig. 1:**
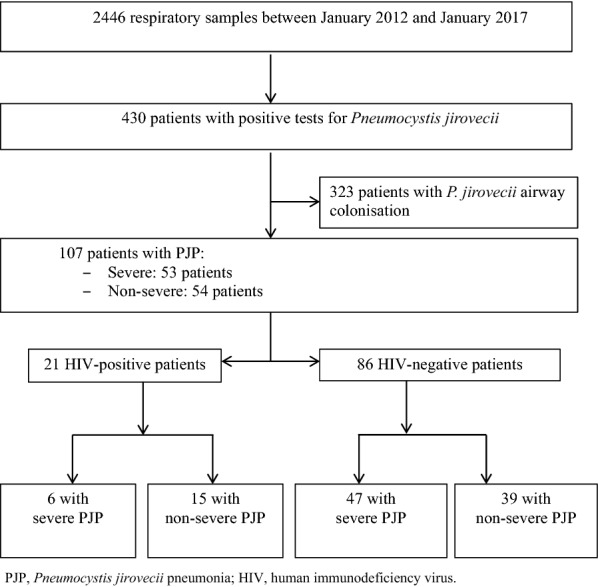
Flow chart. PJP, *Pneumocystis jirovecii* pneumonia; HIV, human immunodeficiency virus

Of the 2446 respiratory samples tested during the study period, 514 from 430 patients were positive for *P. jirovecii*. Of these 430 patients, 107 met our PJP criteria and were included in the study; Table 1 reports their main characteristics. The remaining 323 patients were classified as having bronchial *P. jirovecii* colonisation (Fig. 1). The mean number of patients included per year was 21 and the number of patients per year increased gradually over time to reach a peak of 28 patients in 2015 (Additional file 1: Figure S1).

**Table 1:** Demographic characteristics of the 107 patients with *Pneumocystis jirovecii* pneumonia

	Patients with PJP *n* = 107
Age, years, mean ± SD	56 ± 18
Males, n (%)	65 (61)
BMI, kg/m^2^, mean ± SD	23 ± 4
Serum albumin, g/L, mean ± SD	26.8 ± 6.3
Chronic underlying disease, *n* (%)	
Chronic pulmonary disease	33 (30.8)
Chronic kidney disease	24 (22.4)
Chronic heart failure	9 (8.4)
ICU admission, *n* (%)	51 (47.7)
Severe ARDS, *n* (%)	47 (43.9)
PaO_2_/FiO_2_ on admission, mean ± SD	174 ± 112
SAPS2, mean ± SD	33 ± 13
SOFA score on day 1, mean ± SD	3 ± 3
Cause of immunodeficiency, *n* (%)^a^	
Haematological malignancy	37 (34.6)
Solid organ transplant	27 (25.2)
HIV infection	21 (19.6)
HIV viral load, copies/mL, mean ± SD	317,240 ± 474,037
Systemic disease	13 (12.2)
Solid malignancy	12 (11.2)
Primary immunodeficiency	8 (7.5)
Ongoing immunosuppressive therapy, *n* (%)	83 (77.6)
Ongoing glucocorticoid therapy, *n* (%)	51 (47.7)
Prednisolone-equivalent dosage, mg/day, mean ± SD	17 ± 30
PJP prophylaxis, *n* (%)^b^	21 (19.6)
Time from symptom onset to admission, days, mean ± SD	13 ± 22
Time from symptom onset to initiation of effective therapy, days, mean ± SD	16 ± 19.7
Laboratory findings, mean ± SD	
White blood cells/mm^3^	12.5 ± 26.5
Neutrophils/mm^3^	7.4 ± 13
Lymphocytes/mm^3^	2038.1 ± 7913.5
CRP, mg/L	110.1 ± 96.1
LDH, µKat/L	9.8 ± 5.8
Diagnosis of PJP	
Sputum	16 (15)
Positive PJ PCR	15
PJ visible in smears	3
Bronchoalveolar lavage (BAL) fluid	
N of patients (%)	97 (90.7)
Time from onset to BAL, days, mean ± SD	3 ± 5.5
PJ visible in smears, *n* (%)	49 (45.8)
Alveolitis profile, *n* (%)	31 (29)
Co-infection at PJP diagnosis, *n* (%)	
Viral infection	37 (35)
Bacterial infection	29 (18)
Invasive fungal infection	5 (5)
Respiratory support, *n* (%)	
Mechanical ventilation	30 (28)
Non-invasive ventilation	2 (1.9)
Outcome	
90-day mortality, *n* (%)	29 (27.1)

BMI, body mass index; ICU, intensive care unit; ARDS, acute respiratory distress syndrome; SAPS2, simplified acute physiology score version 2; SOFA score, sequential organ failure assessment score; HIV, human immunodeficiency virus; PJP, *Pneumocystis jirovecii* pneumonia; CRP, C-reactive protein; LDH, lactate dehydrogenase; PJ, *Pneumocystis jirovecii*

^a^The total exceeds 100% because some patients had more than one cause of immunodeficiency

^b^Of these 21 patients, 7 followed their prescribed prophylactic regimen (aerosolised pentamidine, *n* = 6; and atovaquone, *n* = 1) and 14 did not (trimethoprim/sulfamethoxazole, *n* = 11; and aerosolised pentamidine, *n* = 3)

Of the 107 patients with PJP, 97 had positive BAL fluid, by a direct examination of BAL fluid in 49 patients and only detected by PCR in 48 patients. The induced sputum test was positive in 16 patients (by PCR, *n* = 15; and/or Grocott-Gomori stain, *n* = 3). Both the BAL fluid and the induced sputum test were positive in 6 patients. All patients were immunocompromised with haematological malignancy ranking first (*n* = 37, 35%), followed by solid organ transplantation (*n* = 27, 25%), HIV-infection (*n* = 21, 20%), systemic diseases (*n* = 13, 12%), solid tumors (*n* = 12, 11%) and primary immunodeficiency (*n* = 6, 8%). The mean HIV viral load at PJP diagnosis was 317,240 copies for HIV-positive patient. Only 21 (19.6%) patients were given PJP prophylaxis. Of these, 7 were compliant with the prescription during the last 2 months before diagnosis, which never consisted in TMP/SMZ.

The first-line PJP therapy was TMP/SMZ for 100 (93.5%) patients, 6 (5.6%) patients being treated with atovaquone; no PJP therapy was given to the remaining patient, who died within 24 h after ICU admission (PJP diagnosis was made post-mortem). Adjunctive glucocorticoid therapy was given to 61 (57%) patients based on severity criteria. Sixteen patients were switched from TMP-SMZ to atovaquone (*n* = 14) or pentamidine (*n* = 2), due to acute kidney injury (*n* = 9), myelotoxicity (*n* = 5) allergy (*n* = 1), or hepatic cytolysis (*n* = 1); 2 of these 16 patients had both acute kidney injury and myelotoxicity.

Of 97 patients with available BAL fluid cytology results, 31 (32%) had evident alveolitis profile. Mean BAL fluid percentages in the 97 patients were 21.7 ± 21.3 for neutrophils, 46 ± 25.7 for macrophages, and 32 ± 24.7 for lymphocytes. Bacterial co-infection was diagnosed in 19 (18%) patients, viral co-infection in 37 (35%) patients, and fungal co-infection in 5 (5%) patients (Additional file 1: Table S1). Lymphocytes and neutrophils mean ratio were, respectively, 22% and 23% in HIV patients, 34% and 21% in non-HIV patients, 22% and 30% in dead patients, 35% and 19% in survivors patients, 36% and 21% with only PJP patients, 29% and 22% during coinfection. We did not observe in our study eosinophilic and neutrophilic alveolitis.

### Factors associated with severity and 90-day mortality

Of the 107 patients, 53 (49.5%) were classified as severe PJP (Table 2). ICU admission was required in 50 patients, including 30 who received MV. The HIV serology was positive in 6/53 (11%) patients with severe PJP and 15/54 (28%) patients with non-severe PJP.

**Table 2:** Comparison of patients with severe and non-severe *Pneumocystis jirovecii* pneumonia

	Severe PJP (*n* = 53)	Non-severe PJP (*n* = 54)	*p* value
Age, years, mean ± SD	59.23 ± 18	51.97 ± 18	*0.019*
Males, *n* (%)	34 (64)	31 (57)	0.554
BMI, kg/m^2^, mean ± SD	23.55 ± 8	22.45 ± 8	0.254
Serum albumin, g/L, mean ± SD	25.08 ± 12	28.85 ± 12	*<* *0.001*
Chronic underlying disease, *n* (%)			
Chronic pulmonary disease	17 (32.08)	16 (29.63)	0.836
Chronic kidney disease	10 (18.87)	14 (25.93)	0.423
Chronic heart failure	6 (11.32)	3 (5.56)	0.320
ICU admission, *n* (%)	48 (90.57)	3 (5.56)	*<* *0.0001*
PaO_2_/FiO_2_ on admission, mean ± SD	111.38 ± 54	269.66 ± 112	*<* *0.0001*
SAPS2, mean ± SD	38.37±	28.19±	*<* *0.0001*
SOFA score on day 1, mean ± SD	5 ± 3.1	1 ± 1.5	*<* *0.0001*
Cause of immunodeficiency, *n* (%)^a^			
Haematological malignancy	21 (39.6)	16 (29.6)	0.314
Solid organ transplant	13 (24.53)	14 (25.93)	1
HIV infection	6 (11.1)	15 (28.3)	*0.050*
Systemic disease	9 (16.98)	4 (7.41)	0.150
Solid malignancy	6 (11.32)	6 (11.11)	1
Primary immunodeficiency	3 (5.66)	5 (9.26)	0.477
Ongoing immunosuppressive therapy, *n* (%)	45 (84.91)	38 (70.37)	0.104
Ongoing glucocorticoid therapy, *n* (%)	30 (56.6)	21 (38.89)	0.083
Prednisolone-equivalent dosage, mg/day, mean ± SD	20.2 ± 30.26	14.69 ± 30.42	0.102
PJP prophylaxis, *n* (%)	9 (17)	12 (22)	0.495
Time from symptom onset to admission, days, mean ± SD	10.85 ± 20.38	15.2 ± 20.53	0.327
Time from symptom onset to initiation of effective therapy, days, mean ± SD	13.89 ± 21.9	18.04 ± 17.1	0.28
Laboratory findings, mean ± SD			
White blood cells/mm^3^	17.76 ± 36	7.29 ± 8.15	*<* *0.001*
Neutrophils/mm^3^	10.07 ± 18	4.77 ± 4.34	*0.001*
Lymphocytes/mm^3^	2262.48 ± 8766	1809.4 ± 7017	0.766
CRP, mg/L	148.75 ± 105	74.04 ± 70.65	*<* *0.001*
LDH, µKat/L	9.79 ± 7	5.93 ± 3.9	*0.012*
Bronchoalveolar lavage (BAL), time from symptom onset, days, mean ± SD	2.23 ± 4.69	3.27 ± 3.72	0.229
PJ visible in smears, *n* (%)	31 (58.49)	18 (33.33)	*0.011*
Neutrophils, %, mean ± SD	30.09	14.46	*<* *0.001*
Macrophages, %, mean ± SD	43.76	49.7	0.349
Lymphocytes, %, mean ± SD	31.22	34.24	0.383
Alveolitis profile, *n* (%)	9 (16.98)	22 (40.74)	*0.007*
Co-infection at PJP diagnosis			
Viral infection, *n* (%)	26 (49.06)	19 (35.19)	0.173
Bacterial infection, *n* (%)	9 (16.98)	11 (20.37)	0.805
Invasive fungal infection, *n* (%)	2 (3.77)	3 (5.56)	1
Respiratory support			
Mechanical ventilation, *n* (%)	30 (56.60)	0	*<* *0.0001*
Non-invasive ventilation, *n* (%)	2 (3.77)	0	1
90-day mortality, *n* (%)	24 (45)	5 (9)	*<* *0.0001*

BMI, body mass index; ICU, intensive care unit; ARDS, acute respiratory distress syndrome; SAPS2, simplified acute physiology score version 2; SOFA score, sequential organ failure assessment score; HIV, human immunodeficiency virus; PJP, *Pneumocystis jirovecii* pneumonia; CPR, C-reactive protein; LDH, lactate dehydrogenase; PJ, *Pneumocystis jirovecii*

^a^The total exceeds 100% because some patients had more than one cause of immunodeficiency. Significant values are shown in italicface

Early risk factors associated with severity in the univariate analyses were age > 55 years (OR, 2.6; 95% CI 1.12–6.3; *p *< 0.02), albuminemia < 27 g/L (OR, 3.3; 95% CI 1.25–9; *p *< 0.001), blood neutrophil count > 6.5 G/L (OR, 6.5; 95% CI 2.4–20; *p *< 0.001), BAL fluid neutrophils > 12% (OR, 5.7; 95% CI 2–18; *p *< 0.001), *P. jirovecii* oocysts observed at direct examination of BAL fluid (OR, 2.8; 95% CI 1.2–6.9; *p *< 0.01), higher baseline lactic dehydrogenase value (OR, 1.35; 95% CI 1.13–1.68; *p *< 0.002), and higher CRP (OR, 1.01; 95% CI 1.01–1.02; *p *< 0.001). A BAL alveolitis profile was protective (OR, 0.3; 95% CI 0.1–0.8; *p *< 0.01). By multivariate analysis, factors independently associated with severe PJP were older age (OR, 3.36; 95% CI 1.4–8.5; *p *< 0.05), *P. jirovecii* oocysts observed at direct examination of BAL fluid (OR, 1.3; 95% CI 1.54–9.3; *p *< 0.05) and the absence of a BAL fluid alveolitis profile (OR, 3.2; 95% CI 1.27–8.8; *p *< 0.04).

Two factors were independently associated with 90-day mortality by multivariate analysis, a worse SOFA score was associated with higher 90-day mortality (OR, 1.05; 95% CI 1.02–1.09; *p *< 0.001), whereas BAL fluid alveolitis profile was associated with lower 90-day mortality (OR, 0.79; 95% CI 0.65–0.96; *p *< 0.05) (Table 3). HIV serology was a protective factor in univariate analysis but was not statistically associated with protective factor in the multivariate 90-day mortality analysis. In survival analysis HIV patients presenting with PJP was associated with statistically better prognostic than that of patients with hematologic diseases or solid cancer (Additional file 1: Figures S2, S3).

**Table 3:** Risk factors for 90-day mortality in the overall population (patients where BAL was performed, *n* = 85)

	Univariate analysis (*n* = 85)			Multivariate analysis (*n* = 85)		
	OR	95% CI	*p* value	OR	95% CI	*p* value
Characteristics						
Age > 55 years	1.24	1.02–1.5	*<* *0.05*	1.02	0.82–1.2	0.87
BMI	1	0.97–1.02	0.94			
Serum albumin, g/L	0.99	0.97–1.01	0.4			
Glucocorticoid treatment	1.15	0.95–1.4	0.16			
HIV infection	0.74	0.52–0.95	*0.02*	0.8	0.82–1.2	0.87
Bronchoalveolar lavage (BAL)						
Alveolitis profile of BAL	0.75	0.61–0.92	*0.007*	0.79	0.65–0.96	*0.02*
Neutrophils	1.02	0.99–1.04	0.12			
Macrophages	1.01	0.99–1.04	0.21			
Lymphocytes	0.97	0.94–0.99	0.06			
Co-infection						
Bacterial	1.2	0.95–1.6	0.13			
Viral	1.25	1.02–1.52	*0.03*	1.2	0.99–1.4	0.07
Invasive fungal	1.21	0.76–1.9	0.41			
Blood tests						
White blood cell counts	1	0.99–1	0.69			
Polynuclear neutrophils	1.07	0.8–1.4	0.76			
Lymphocytes	0.95	0.8–1.4	0.62			
Serum CRP	1	0.99–1	0.24			
Severity						
SAPS2	1	1–1.01	*0.008*			
SOFA score	1.08	1.05–1.1	*<* *0.0001*	1.05	1.02–1.09	*0.0002*
Severe SDRA	1.38	1.14–1.7	*<* *0.0001*			
P/F ratio > 200	0.78	0.64–0.96	*<* *0.05*			

Italics characters correspond to the analysis parameters with a statistically significant difference

BMI, body mass index; SAPS2, simplified acute physiology score version 2, SOFA score, sequential organ failure assessment score; HIV, human immunodeficiency virus

In the subgroup of HIV-negative patients, similar findings were obtained, then viral co-infection were independently associated with higher 90-day mortality (OR, 1.25; 95% CI 1.02–1.55; *p *< 0.05) (Additional file 1: Tables S2, S3). Factors associated with 90-day mortality in ICU patients were SOFA score and non-HIV patients (Additional file 1: Table S4).

## Discussion

In this prospective study, over four-fifths of PJP patients were HIV-negative, and half met our criteria for severe disease. A worse SOFA score on admission and viral co-infection were independently associated with higher 90-day mortality in both whole patients and HIV-negative patients. Importantly, a BAL fluid cytological profile consistent with alveolitis was associated with lower 90-day mortality.

Severe PJP on admission as defined for our study was associated with a 56% risk of receiving MV, in keeping with recent results from large cohort studies [3, 23]. Given the prognostic significance of severity, an improved knowledge of early risk factors for severity is helpful to identify patients requiring more intensive monitoring and treatment.

As illustrated here, HIV patients less often experienced severe PJP compared to their HIV-negative counterparts, in agreement with earlier data [5, 8, 24–27]. The reduced severity of PJP in HIV-positive patients is more likely to be associated with the particularity of HIV-induced immunosuppression, of which pneumocystis is a hallmark of a severe adaptive cellular impairment. The co-morbidities in non-HIV patients (onco-hematology, solid organ transplantation and system diseases) may also contribute to their greater vulnerability. The immune recovery allowed by the initiation of antiretroviral therapy is probably correlated with better long-term outcomes in HIV-positive patients than in non-HIV patients whose profound immunosuppression is more frequently extended. HIV-positive patients also have lower neutrophil counts in BAL fluid samples [28], are less likely to develop severe PJP, and have lower mortality rates compared to HIV-negative patients [11, 26–29]. The SOFA score, viral co-infection and absence of alveolitis remained independently associated with a 90-day higher mortality in HIV-negative patients, suggesting that it is a strong independent marker in the whole PJP population.

By univariate analysis, early risk factors for severity in HIV-negative patients were markers for vulnerability (older age and lower serum albumin) and for inflammation (systemic inflammatory syndrome characterized by blood and alveolar polynucleosis serum CRP level). The patient subgroup at the most severe end of the spectrum had the highest CRP levels and neutrophil counts, suggesting that anti-inflammatory treatments might improve patient outcomes. Our study suggests that future prospective studies on adjunctive treatments for PJP should focus on HIV-negative patients, notably solid organ and haematological stem cell transplant recipients, meeting criteria for severe PJP (e.g., SOFA score > 2 with a low PaO_2_/FiO_2_ ratio). The potential relevance of a BAL fluid cytology profile consistent with alveolitis should probably also be taken into account when assessing the efficacy of new treatment regimens.

As expected, mortality within the first 90 days was significantly higher in patients with severe versus non-severe PJP. The independent association between a worse SOFA score at admission and 90-day mortality confirms the appropriateness of an evaluation with an intensivist to consider ICU admission of patients with oxygen-dependent PJP [30]. The quickSOFA, which is a simplified score based on three criteria, is easy to determine by non-intensivists and may be useful for determining when advice from an intensivist should be sought [31]. In addition to a worse SOFA score, viral co-infection at the time of PJP diagnosis was associated with higher 90-day mortality. Viral infections consisted mostly (67%) in reactivation of latent viruses (cytomegalovirus, herpes simplex virus, or Epstein-Barr virus), suggesting that this subgroup may have been characterised by a more profound immune deficiency. Taken together, this finding supports the hypothesis that the outcome of PJP is also closely related to the underlying diagnosis and immune response.

An important finding from this study is the clear association between a BAL fluid cytology profile consistent with alveolitis (> 10% lymphocytes, > 5% neutrophils, and presence of activated macrophages) and less severe PJP and lower 90-day mortality. The presence of neutrophils in BAL fluid has often been noted in clinical and experimental studies of acute respiratory distress syndrome (ARDS) [32, 33]. Three patient subgroups can be identified in our population, one defined by alveolitis, the other one PJP occuring in HIV-positive individuals, both having a better prognosis and the last one defined by a SOFA score above 2 on admission who have a more severe prognosis. Taken together, these results suggest that specific immunological characteristics might allow the identification of patient subgroups with different treatment needs and outcomes.

CD4^+^ T cell levels were non-significantly higher in patients with alveolitis, whereas glucocorticoid exposure was comparable in the two groups. To our knowledge, alveolitis during PJP has not been described as a good prognostic factor yet [21]. In murine models of CD4^+^ T-cell depleted mice, increased alveolar recruitment of CD8^+^ T cells induced by interleukin-7 therapy improved *P. jirovecii* clearance [34]. Lymphocyte count in BAL fluid is increased in patients with alveolitis, which was associated with a better prognosis in our study. Thus, alveolitis during PJP might reflect maintenance of an effective pulmonary immune response including CD8^+^ T-cell recruitment. Glucocorticoid therapy might, therefore, be unnecessary in patients with PJP and alveolitis, although their specific response to glucocorticoids has not been investigated to date.

Four-fifths of our patients had not been prescribed PJP prophylaxis, and among those with a prescription only one-third were compliant. These findings confirm earlier results [16, 35] and further identify the absence of TMP/SMZ prophylaxis as a major risk factor for PJP in high-risk patients [29]. Providing appropriate prophylactic anti-microbial treatments to patients with immunosuppression, notably related to haematological diseases and transplantation, is crucial to improve patient outcomes. Adherence to prophylactic treatment must be supported at each follow-up visit. PJP usually develops in patients with CD4^+^counts below 300/mm^3^ [36–38], although the depth of lymphopenia does not correlate with PJP severity [39, 40]. A history of glucocorticoid exposure is an often reported risk factor for PJP in HIV-negative patients [41, 42]. Among our patients, over half were receiving glucocorticoid therapy at the diagnosis of PJP, in a mean prednisone-equivalent dosage of 20 mg/day. Adjuvant glucocorticoid therapy was given to most severe patients who failed antibiotic treatment alone. Given the history of glucocorticoid exposure, the effects of adjuvant glucocorticoid therapy were difficult to assess. The role for adjuvant glucocorticoid therapy in patients with PJP is debated [26, 43–47] and is currently being assessed in a prospective study (ClinicalTrials.gov Identifier: NCT02944045).

Serum LDH level at PJP diagnosis was associated with poor outcomes in HIV-positive and HIV-negative patients in several studies [48]. In our population, LDH > 7 µkat/L (420 U/L) levels were associated with severe PJP by univariate analysis but not with higher 90-day mortality by multivariate analysis. Missing data or a role for unidentified confounders may explain this result.

A limitation of our study is the use of non-validated criteria for defining severe PJP. The European Conference on Infections in Leukaemia-5 group has defined severe PJP, like those defined for HIV-positive patients [49] such as PaO_2_ less than 8 kPa, oxygen saturation less than 91%, and radiological impairment. These criteria were only used in retrospective study of PJP patients [45]. However, these criteria have not been prospectively validated in non-HIV patients. HIV-patients less often experienced severe PJP compared to their HIV-negative counterparts, enhancing the need to propose an early severity scale adapted to this specific population. We, therefore, relied on criteria indicating hypoxemic ARF, namely, FiO_2_ ≥ 50% or NIV or MV. The strong association of severe PJP with higher 90-day mortality indicates that our definition successfully identified severe PJP.

## Conclusion

In a large prospective cohort of patients admitted for PJP, the SOFA score on admission and presence of viral co-infection were independently associated with higher 90-day mortality. Importantly, a BAL fluid cytology profile suggesting alveolitis was associated with less severe PJP and lower 90-day mortality. Our findings, notably the associations linking the SOFA score and alveolitis to 90-day mortality, deserve further evaluation in a multicentre prospective study. Future studies of adjunctive treatments for PJP therapy should differentiate between patients with and without alveolitis.

## Supplementary information

**Additional file 1.** Additional figures and tables.

## Data Availability

The datasets used and/or analysed during the current study are available from the corresponding author on reasonable request.

## Abbreviations

ARDS: severe acute respiratory distress syndrome
BAL: broncho-alveolar lavage
BMI: body mass index
CI: confidence interval
CMV: cytomegalovirus
CRP: C-reactive protein
EBV: Epstein–Barr virus
FiO_2_: fraction of inspired oxygen
HIV: human immunodeficiency virus
HSV1: herpes simplex virus
ICU: intensive care unit
LDH: lactate dehydrogenase
MV: invasive mechanical ventilation
NIV: non-invasive ventilation
OR: odds ratio
PaO_2_: arterial partial pressure of oxygen
PCR: real-time polymerase chain reaction
PJP: *Pneumocystis jirovecii* pneumonia
RSV: respiratory syncytial virus
SAPS2: simplified acute physiology score version 2
SD: standard deviation
SOFA score: sequential organ failure assessment score
TMP-SMX: trimethoprim–sulfamethoxazole
