# Supplementary material for: Outcome and prognostic factors of Pneumocystis jirovecii pneumonia in immunocompromised adults: a prospective observational study
Source: Ann Intensive Care. 2019 Nov 27;9:131. doi: 10.1186/s13613-019-0604-x (PMC6881486; doi:10.1186/s13613-019-0604-x)
Supplement: Supplementary file 1 — Additional file 1. Additional figures and tables. [file 13613_2019_604_MOESM1_ESM.docx]

**Additional material**

**Figure S1:** Changes over time in the numbers of patients with *Pneumocystis jirovecii* pneumonia per year over the study period, according to HIV status.

HIV, human immunodeficiency virus

**Table S1**: Co-infection at the diagnosis of *Pneumocystis jirovecii* pneumonia in the population of 107 patients

|  | Patients with co-infection, n (%) |
| --- | --- |
| **Bacterial infection** | 19 (18%) |
| *Pseudomonas aeroginosa* | 4 (21%) |
| *Staphylococcus aureus* | 4 (21%) |
| *Escherichia coli* | 3 (20%) |
| *Streptococcus spp.* | 2 (16%) |
| *Serratia spp.* | 1 (5%) |
| *Haemophilus influenzae* | 1 (5%) |
| **Viral infection** | 37 (35%) |
| *Cytomegalovirus* | 25 (68%) |
| *Epstein-Barr virus* | 6 (16%) |
| *Rhinovirus* | 6 (16%) |
| *Herpes simplex virus 1* | 4 (11%) |
| *Influenzae* | 3 (8%) |
| *Parainfluenzae* | 2 (5%) |
| *Adenovirus* | 1 (3%) |
| *Enterovirus* | 1 (3%) |
| *Normetavirus* | 1 (3%) |
| *Respiratory syncytial virus* | 1 (3%) |
| *Coronavirus* | 1 (3%) |
| **Other invasive infections** | 5 (5%) |
| *Aspergillus fumigatus* | 3 (60%) |
| *Toxoplasma gondii* | 2 (40%) |
|  |  |
|  |  |
|  |  |
|  |  |

Among 5 HIV-positive patients with co-infection, 1 had *Toxoplasma gondii*infection, 1 had *Streptococcus pneumoniae*infection, and 3 had cytomegalovirus infection (viremia in 2 and positive broncho-alveolar lagage fluid in 1); 1 of these 5 patients had both *T. gondii*and cytomegalovirus infection.

**Table S2**: Comparison of HIV-negative patients with non-severe versus severe *Pneumocystis jirovecii* pneumonia

|  | Severe PJP  (n=47) | Non-severe PJP (n=39) | *p* value |
| --- | --- | --- | --- |
| **Age, years, mean±SD** | 61.6 ± 17.5 | 54.6 ± 18.2 | **0.045** |
| **Males, n (%)** | 30 (63.8) | 19 (48.7) | 0.192 |
| **BMI, Kg/m^2^, mean±SD** | 23.6 ± 4.3 | 23.5 ± 4.26 | 0.988 |
| **Serum albumin , g/L, mean±SD** | 25.1 ± 6 | 29.48 ± 5.74 | **0.006** |
| **Chronic underlying disease, n (%)** |  |  |  |
| Chronic pulmonary disease | 9 (19.1) | 14 (35.9) | 0.093 |
| Chronic kidney disease | 17 (36.2) | 16 (41) | 0.663 |
| Chronic heart failure | 6 (12.8) | 2 (5.1) | 0.283 |
|  |  |  |  |
| **ICU admission, n (%)** | 42 (89.4) | 2 (5.1) | **<0.0001** |
| **PaO_2_/FiO_2_ on admission, mean±SD** | 108.2 ± 51.8 | 244.8 ± 103.2 | **<0.0001** |
| **SAPS2, mean±SD** | 38.2 ±15.3 | 27.7 ± 10.2 | **0.001** |
| **SOFA score on day 1, mean±SD** | 5.4 ± 3.2 | 1.8 ± 1.6 | **<0.0001** |
| **Cause of immunodeficiency, n (%)^a^** |  |  |  |
| Haematological malignancy | 20 (42.5) | 16 (41) | 1.000 |
| Solid organ transplant | 13 (27.7) | 14 (35.9) | 0.487 |
| HIV infection | 0 | 0 | 1.000 |
| Systemic disease | 9 (19.1) | 4 (10.3) | 0.366 |
| Solid malignancy | 6 (12.8) | 6 (15.4) | 0.763 |
| Primary immunodeficiency | 3 (6.4) | 5 (12.8) | 0.457 |
| Ongoing immunosuppressive therapy, n (%) | 45 (95.7) | 38 (97.4) | 1.000 |
| Ongoing glucocorticoid therapy, n (%) | 30 (63.8) | 21 (53.8) | 0.384 |
| Prednisolone-equivalent dosage, mg /d | 22.8 ±31.9 | 20.3 ± 31.1 | 0.453 |
| **PJP prophylaxis, n (%)** | 9 (19.1) | 10 (25.6) | 1.000 |
| **Time from symptom onset to admission, days, mean±SD** |  |  |  |
|  | 12.46 | 11.54 | 0.315 |
| **Laboratory findings, mean±SD** |  |  |  |
| White blood cells/mm^3^ | 17.5 ±37.4 | 8 ± 9.4 | **0.011** |
| Neutrophils/mm^3^ | 10.4± 18.6 | 5.1 ± 4.7 | **0.010** |
| Lymphocytes/mm^3^ | 1072 ± 1605 | 2264.1 ± 8305 | 0.928 |
| CRP, mg/L | 155.4 ±102 | 94.1 ± 74.3 | **0.006** |
| LDH, µKat/L | 8.7± 4.9 | 6± 4.2 | **0.049** |
| **Bronchoalveolar lavage (BAL)** |  |  |  |
| Time from onset to BAL, days, mean±SD | 12.5 | 11.5 | 0.315 |
| PJ visible in smears, n (%) | 27 (57.4) | 8 (20.5) | **0.001** |
| Neutrophils in BAL, %, mean±SD | 30 | 12.7 | **0.001** |
| Macrophages in BAL %, mean±SD | 42.4 | 48.5 | 0.302 |
| Lymphocytes in BAL, %, mean±SD | 33.2 | 37.1 | 0.307 |
| Alveolitis profile, n (%) | 8 (17) | 19 (48.7) | **0.003** |
| **Co-infection at PJP diagnosis, n (%)** |  |  |  |
| Viral infection | 22 (46.8) | 12 (30.8) | 0.184 |
| Bacterial infection | 8 (17) | 10 (25.6) | 0.426 |
| Invasive fungal infection | 2 (4.3) | 2 (5.1) | 1.000 |
| **Respiratory support** |  |  |  |
| Mechanical ventilation, n (%) | 28 (59.6) | 0 | **<0.0001** |
| Non-invasive ventilation, n (%) | 2 (4.3) | 0 |  |
|  |  |  |  |
| **Day-90 mortality, n (%)** | 24 (51) | 4 (10) | **<0.0001** |
|  |  |  |  |
|  |  |  |  |

^a^The total exceeds 100% because some patients had more than one cause of immunodeficiency.

**Table S3**: Risk factors for 90-day mortality in the group of 75 HIV-negative patients

|  | Univariate analysis | | | Multivariate analysis | | |
| --- | --- | --- | --- | --- | --- | --- |
|  | OR | 95%CI | *p* value | OR | 95%CI | *p* value |
| Characteristics |  |  |  |  |  |  |
| - Age>55 years - BMI | 1.1  0.99 | 0.87-1.4  0.96-1.01 | 0.4  0.4 |  |  |  |
| - Serum Albumin, g/L | 0.99 | 0.97-1 | 0.3 |  |  |  |
| - Glucocorticoid treatment before the PJP diagnosis | 1.03 | 0.8-1.3 | 0.6 |  |  |  |
|  |  |  |  |  |  |  |
| Alveolitis profile in BAL fluid | 0.7 | 0.56-0.88 | 0.003 | 0.77 | 0.62-0.96 | 0.02 |
| Co-infection |  |  |  |  |  |  |
| - Bacterial | 1.13 | 0.87-1.48 | 0.4 |  |  |  |
| - Viral | 1.35 | 1.09-1.7 | 0.008 | 1.25 | 1.02-1.55 | 0.03 |
| - Invasive fungal | 1.4 | 0.8-2.4 | 0.3 |  |  |  |
| Laboratory results |  |  |  |  |  |  |
| - White blood cell counts - Polynuclear neutrophils - Lymphocytes | 1  1.04  0.96 | 0.99-1  0.8-1.4  0.8-1.2 | 0.9  0.75  0.66 |  |  |  |
| - Serum CRP level | 1 | 0.99-1 | 0.94 |  |  |  |
| Severity |  |  |  |  |  |  |
| - SAPS2 - SOFA score - Severe SDRA - P/F ratio>200 | 1.01  1.07  1.4  0.79 | 1.01-1.02  1.04-1.7  1.09-1.7  0.62-1 | **0.02**  <0.0001  0.008  0.06 | corr  1.05  Corr  corr | 1.02-1.09 | 0.005 |
|  |  |  |  |  |  |  |
|  |  |  |  |  |  |  |

OR, odds ratio; 95%CI, 95% confidence interval; BMI, body mass index; HIV, human immunodeficiency virus; BAL, broncho-alveolar lavage; CRP, C-reactive protein; SOFA score, Sequential Organ Failure Assessment score

CORR= colinearity is too high to include both the parameters in the multivariate analysis, thus the weakest association is excluded from the model.

**Table S4**: Risk factors for 90-day mortality in the ICU population (n=49)

|  | Univariate analysis (n=49) | | |
| --- | --- | --- | --- |
|  | OR | 95%CI | *p* value |
| Characteristics |  |  |  |
| - Age>55 years - BMI | 1.3  0.99 | 0.98-1.7  0.95-1.02 | 0.07  0.7 |
| - Serum albumin, g/L | 1 | 0.98-1.03 | 0.84 |
| - Glucocorticoid treatment - HIV infection | 1.24  **0.63** | 0.94-1.6  **0.42-0.94** | 0.13  **0.03** |
| Bronchoalveolar lavage (BAL) |  |  |  |
| - Alveolitis profile of BAL - Neutrophils - Macrophages - Lymphocytes | 0.72  1  1  0.99 | 0.51-1  0.99-1.01  0.99-1.01  0.98-1 | 0.06  0.18  0.34  0.11 |
| Co-infection |  |  |  |
| - Bacterial - Viral | 1.3  1.16 | 0.9-2  0.88-1.5 | 0.18  0.31 |
| - Invasive fungal | 1.32 | 0.74-2.35 | 0.36 |
| Blood tests |  |  |  |
| - White blood cell counts - Polynuclear neutrophils - Lymphocytes - Serum CRP | 1  1  0.96  1 | 0.99-1  0.73-1.4  0.72-1.21  0.99-1 | 0.75  0.94  0.94  0.7 |
| Severity |  |  |  |
| - SAPS2 - SOFA score - Severe SDRA | 1.01  **1.1**  **1.4** | 1-1.02  **1.07-1.15**  **0.98-2** | 0.05  **<0.0001**  **0.07** |
|  |  |  |  |

BMI, body mass index; SAPS2, Simplified Acute Physiology Score version 2, SOFA score, Sequential Organ Failure Assessment score; HIV, human immunodeficiency virus.

**Figure S2:** survival analysis HIV-positive patients compared non-HIV patients presenting with PJP.

HIV, human immunodeficiency virus

**Figure S3:** Description of the mortality rate, related to the severity, HIV status and adjuvant treatment use by steroids.

**With Adjunctive steroid**

**All patients n=20 (37%)**

**Non HIV n=16 (41%)**

Mortality Non HIV patients:

**Mortality D90 n=1(6,3%)**

**Mortality PJP n=0**

***Pneumocystis* pneumonia(n=107)**

**Severe(=53)**

**- All patients(n=53)**: Mortality D90 n=24(45%)

**- Non-HIV(n=47)**: Mortality D90 n=24(51%)

**Without Adjunctive steroid**

**All patients n:12 (22,6%)**

**Non HIV n=12 (25,5%)**

Mortality Non HIV patients:

**Mortality D90 n=9(19,1%)**

**Mortality PJP n=9(19,1%)**

**Without Adjunctive steroid**

**All patients n=34 (63%)**

**Non HIV n=23 (59%)**

Mortality Non HIV patients:

**Mortality D90 n=3(13%)**

**Mortality PJP n=1(8,7%)**

**With Adjunctive steroid**

**All patients n=41 (79,2%)**

**Non HIV n=35 (74,5%)**

Mortality Non HIV patients:

**Mortality D90 n=15(42%)**

**Mortality PJP n=13(37%)**

**Non-Severe(n=54)**

**All patients(n=54)**: Mortality D90 n=5(9,3%)

**Non-HIV(n=39)**: Mortality D90 n=4(10,3%)

HIV, human immunodeficiency virus
